# Supplementary material for: Gradual Distance Dispersal Shapes the Genetic Structure in an Alpine Grasshopper
Source: Genes (Basel). 2019 Aug 5;10(8):590. doi: 10.3390/genes10080590 (PMC6724060; doi:10.3390/genes10080590)
Supplement: Supplementary file 1 [file genes-10-00590-s001.pdf]

Supplementary Materials: Gradual Distance Dispersal Shapes the Genetic Structure in an alpine grasshopper

Juan Carlos Illera, Miguel Arenas, Carlos A. López-Sánchez, José Ramón Obeso, Paola Laiolo

Spatially explicit computer simulations with SPLATCHE2

SPLATCHE3 (Currat et al. 2019) simulates genetic data in the following three steps. First, the program performs a forward in time simulation (Carvajal-Rodriguez 2010, Arenas 2012) of the demography of the whole population over the space. The simulation considers a lattice of demes (given by a map obtained with a Geographic Information System, GIS), and diverse demographic parameters such as population size at the onset of the range expansion, population growth rate, migration rate and carrying capacity. A deme is chosen to start the range expansion, and migration events occur towards neighboring demes under a stepping-stone migration model (Kimura and Weiss 1964) that we denominate here as a gradual-distance dispersal (GDD) model. In addition to the migration rate, the number of emigrants and immigrants depends on the local and departure deme sizes, respectively. Intra-deme demography is modeled by the population growth rate (Currat et al. 2004). Carrying capacity refers to the available resources and is considered to model environmental conditions, it also affects the population growth rate (Currat et al. 2004). Simulations under a model of long-distance dispersal (LDD) are based on Ray and Excoffier (2010). This model considers the proportion of individuals migrating under LDD, the direction of migration events is chosen at random and the migration distance of a LDD event is sampled from a gamma distribution that can be truncated with a maximum distance of dispersal. After this step, the simulation produces the evolutionary history of all the individuals of the entire population over the space and time. In a second step, the coalescent history of a user-specified sample can be reconstructed considering the history of the entire population previously obtained. This is a backwards in time simulation of the evolutionary history of the sample until its most recent common ancestor (MRCA). In a third step, the program simulates molecular evolution along the coalescent tree of the sample from a random sequence assigned to the MRCA and by introducing substitution events along branches according to a substitution model of evolution (Yang 2006).

Table 1. Genetic diversity statistics of *Chorthippus cazurroi* per population. N: sample size. H: number of haplotypes. Hd: haplotype diversity. Std (Hd): Standard deviation of Hd. Pi: nucleotide diversity per site. Std (Pi): Standard deviation of Pi (Nei 1987). θ: Theta ($2N_e\mu$) per site from number of variable sites (Tajima 1996). Group: localities grouped according to their geographic distribution (Figure S1).

Locality	Acronym	N	H	Hd	Std (Hd)	Pi	Std (Pi)	θ	Group	Location
Cantu l'Osu	COsu	20	3	0.574	0.055	0.0011	0.0002	0.001	1	Central
Campigüeños	Cam	11	3	0.345	0.172	0.0006	0.0003	0.0012	2	Western
Llambria	Lla	11	5	0.818	0.083	0.0022	0.0005	0.0024	2	Western
Tiatordos	Tia	16	8	0.892	0.048	0.0043	0.0005	0.0037	2	Western
Maciédome	Mac	18	2	0.425	0.099	0.0007	0.0002	0.0005	2	Western
Peña Ten	Ten	15	3	0.257	0.142	0.0022	0.0013	0.0037	3	Western
Pileñes	Pil	10	1	0	0	0	0	0	3	Western
Cantu Cabroneru	CC	15	3	0.257	0.142	0.0005	0.0003	0.0011	4	Central
Traviesos	Tra	15	3	0.648	0.088	0.0021	0.0004	0.0016	5	Central
Cotalba	Cot	15	2	0.248	0.131	0.0004	0.0002	0.0005	5	Central
Vega Ario	Va	15	3	0.362	0.145	0.0011	0.0005	0.0016	5	Central
Vega Huerta	Vh	16	3	0.242	0.135	0.0006	0.0004	0.0011	5	Central
Vegarredonda	VR	17	1	0	0	0	0	0	5	Central
Tiros Navarros	NV	25	4	0.23	0.11	0.0013	0.0007	0.0023	6	Eastern
Peña Castil	PC	16	4	0.617	0.096	0.0015	0.0004	0.0021	6	Eastern
Liordes	Lio	6	3	0.6	0.215	0.0017	0.0008	0.0023	6	Eastern
Urriellu	U	19	6	0.772	0.062	0.0021	0.0004	0.002	6	Eastern
Camburero	Camb	3	2	0.667	0.314	0.0023	0.0011	0.0023	6	Eastern
Morra Lechugales	MoHie	19	3	0.444	0.124	0.0012	0.0004	0.0015	7	Eastern
Andara	A	15	3	0.257	0.142	0.0005	0.0003	0.0011	7	Eastern
Casetón Andara	Ba	15	2	0.343	0.128	0.0006	0.0002	0.0005	7	Eastern
Rasa	Ras	15	3	0.362	0.145	0.0007	0.0003	0.0011	7	Eastern

Table 2. Prior distributions for both population genetic and environmental parameters applied in the ABC analyses. Despite the prior distributions were oriented by bibliographic information available (see below, source references), we applied a uniform distribution for every prior to allow a well-distributed sampling within the range of the prior.

**Parameter**	**Distribution**	**Source references**
Time of the onset of the expansion [1]	U[15000–20000]	Since the LGM
Population size at the onset of the expansion	U[100–1000]	-
Population growth rate	U[0.2–0.9]	Richards & Waloff (1954)
Migration rate	U[0.05–0.3]	Mason et al. (1995)<br>Nakamura et al. (1964)
Carrying capacity	U[100–1000]	Alves et al. (2016)<br>Arenas et al. (2012,2013)
Mutation rate	U[1.0×10^{-14}–1.0×10^{-5}]	Shapiro et al. (2006)
LDD proportion	U[1.0×10^{-3}–0.05]	Alves et al. (2016)

[1] Time shown in generations (generation time assumed as 1 year).

Table S3. Power to distinguish between the migration models of gradual-distance dispersal (GDD) and long-distance dispersal (LDD), using four ABC approaches: Pr: Pritchard's approach; Rrej: rejection; Rreg: multiple regression; Rnn: neuralnet method rejection. The last three approaches are implemented in the *abc* library of R (Csillery et al. 2012). Rows highlighted in grey show the probability of identifying the true model.

**Model**	**GDD**				**LDD**			
**ABC approach**	**Pr**	**Rrej**	**Rreg**	**Rnn**	**Pr**	**Rrej**	**Rreg**	**Rnn**
**GDD**	0.81	0.93	0.88	0.9	0.16	0.08	0.1	0.12
**LDD**	0.19	0.07	0.12	0.1	0.84	0.92	0.9	0.88

Table S4. Estimates of population genetic and environmental parameters from the real data under the best fitting model. The estimations were performed with the rejection (Rrej), regression (Rreg) and neuralnet (Rnn) approaches implemented in the abc library of R (Csillery et al. 2012). A selection of these results considering the power of the parameters estimation is shown in Table 3.

Parameter	Mode	Mean	Median	90% HPDI [1]
**Rejection approach**				
Time of the onset of the expansion [2]	19,336	17,547	17,558	15,256–19,775
Ancestral population size	821	575	588	157–960
Population growth rate	0.349	0.536	0.527	0.231–0.865
Migration rate	0.072	0.138	0.121	0.055–0.270
Carrying capacity	122	177	154	105–313
Mutation rate	1.37×10^{-06}	4.78×10^{-06}	4.66×10^{-06}	5.15×10^{-07}–9.43×10^{-06}
**Regression approach**				
Time of the onset of the expansion [2]	18,942	18,712	18,723	18,238–19,150
Ancestral population size	918	859	873	719–948
Population growth rate	0.283	0.278	0.278	0.266–0.287
Migration rate	0.017	0.017	0.017	0.016–0.019
Carrying capacity	90	92	91	86–99
Mutation rate	2.88×10^{-08}	3.62×10^{-07}	1.09×10^{-07}	3.10×10^{-10}–1.59×10^{-06}
**Neuralnet approach**				
Time of the onset of the expansion [2]	19,358	18,561	18,574	16,599–20,510
Ancestral population size	662	638	649	540–706
Population growth rate	0.334	0.432	0.430	0.248–0.622
Migration rate	0.091	0.105	0.102	0.076–0.142
Carrying capacity	112	131	126	98–181
Mutation rate	2.06×10^{-07}	2.18×10^{-07}	2.16×10^{-07}	1.86×10^{-07}–2.53×10^{-07}

[1] 90% HPDI indicates the 90% highest posterior density interval. [2] Time shown in generations (generation time assumed as 1 year).

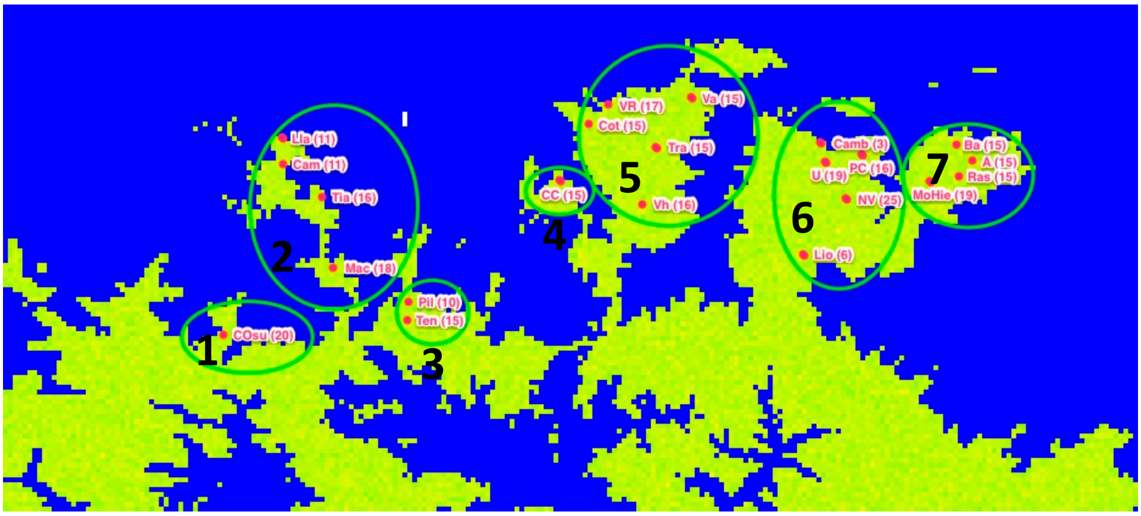

Figure S1. Geographic location of *Chorthippus cazurroi* samples, sample sizes (in brackets) and geographic groups (into green circles) designed to perform the ABC analyses. Black numbers correspond with groups assigned in Table 1. Demes below 1400 m are colored in blue and demes above 1400 m are colored in green. See Table 1 for visualizing the full name of sample locations.

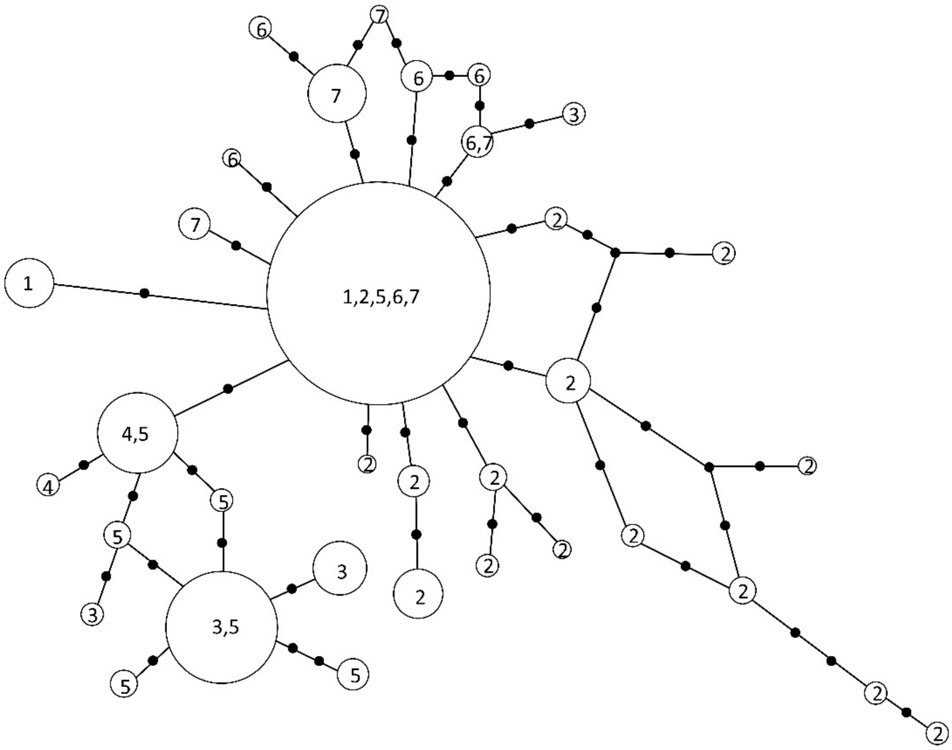

Figure S2. Parsimony network of *Chorthippus cazurroi* in the Cantabrian Mountains based on the cytochrome oxidase subunit I (COI). Black dots depict one-step mutation edge. The size of haplotypes (circles) represents its abundance. Numbers into circles indicate the groups of localities where haplotypes were found. Group 1: COsu. Group 2: Cam, Lla, Tia, Mac. Group 3: Ten, Pil. Group 4: CC. Group 5: Tra, Cot, Va, Vh, VR. Group 6: NV, PC, Lio, U, Camb. Group 7: MoHie, A, Ba, Ras. Acronyms of localities as shown in Table 1.

(A) Rejection approach

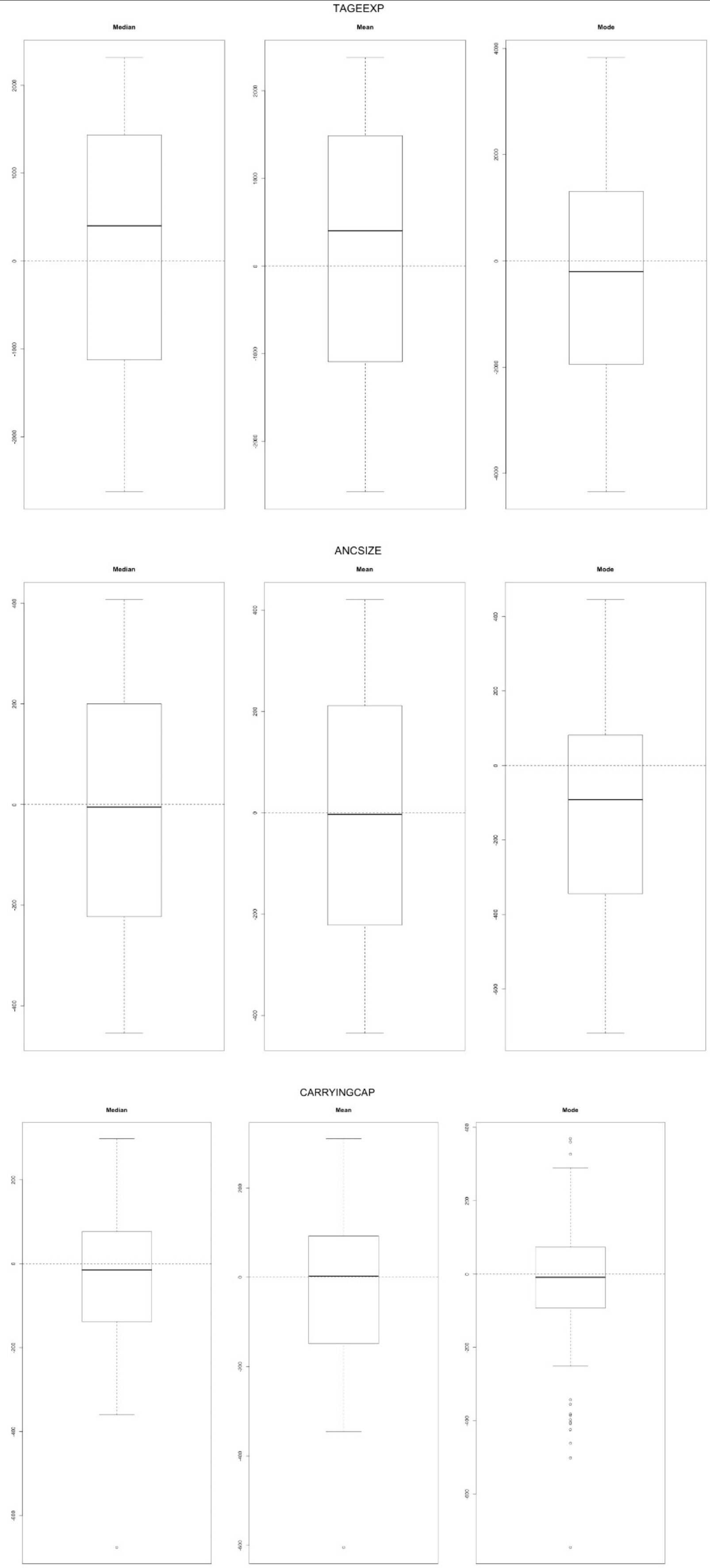

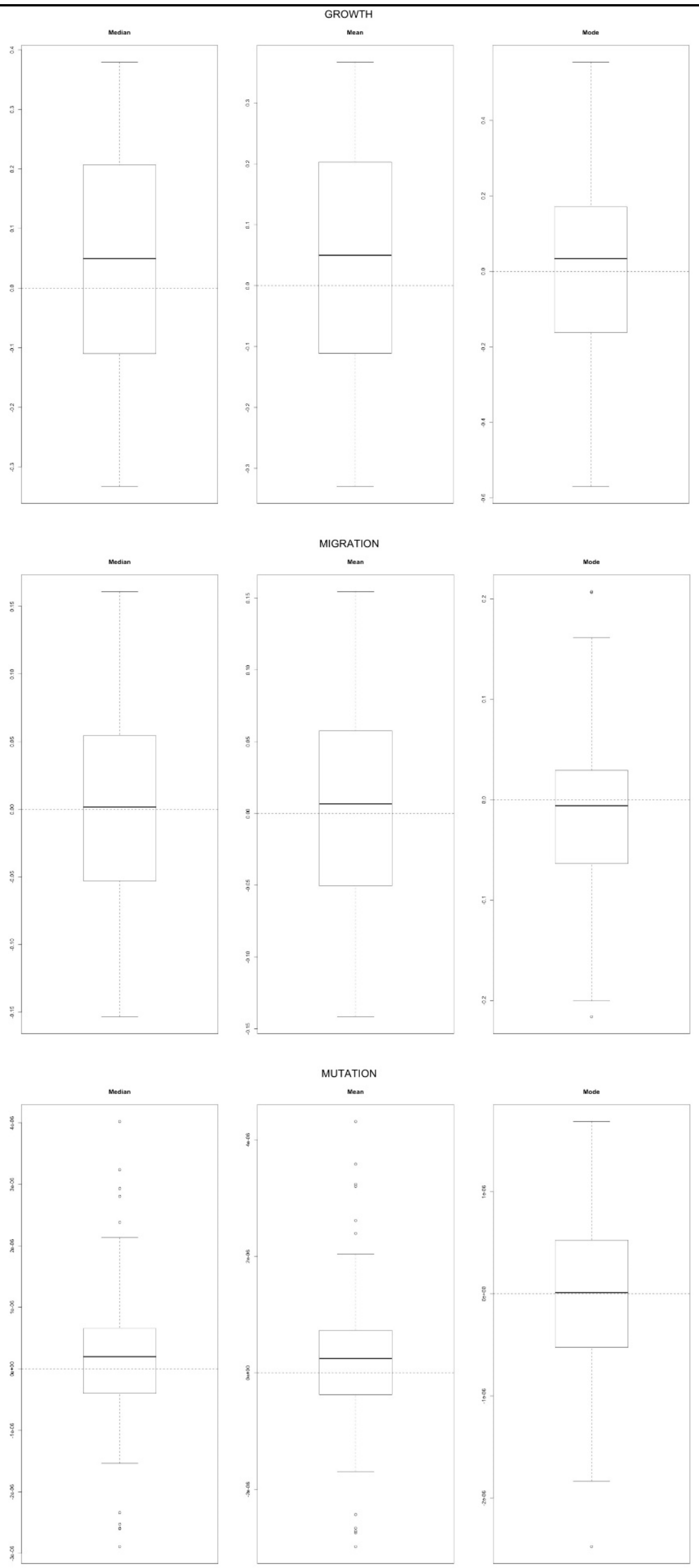

(B) Regression approach

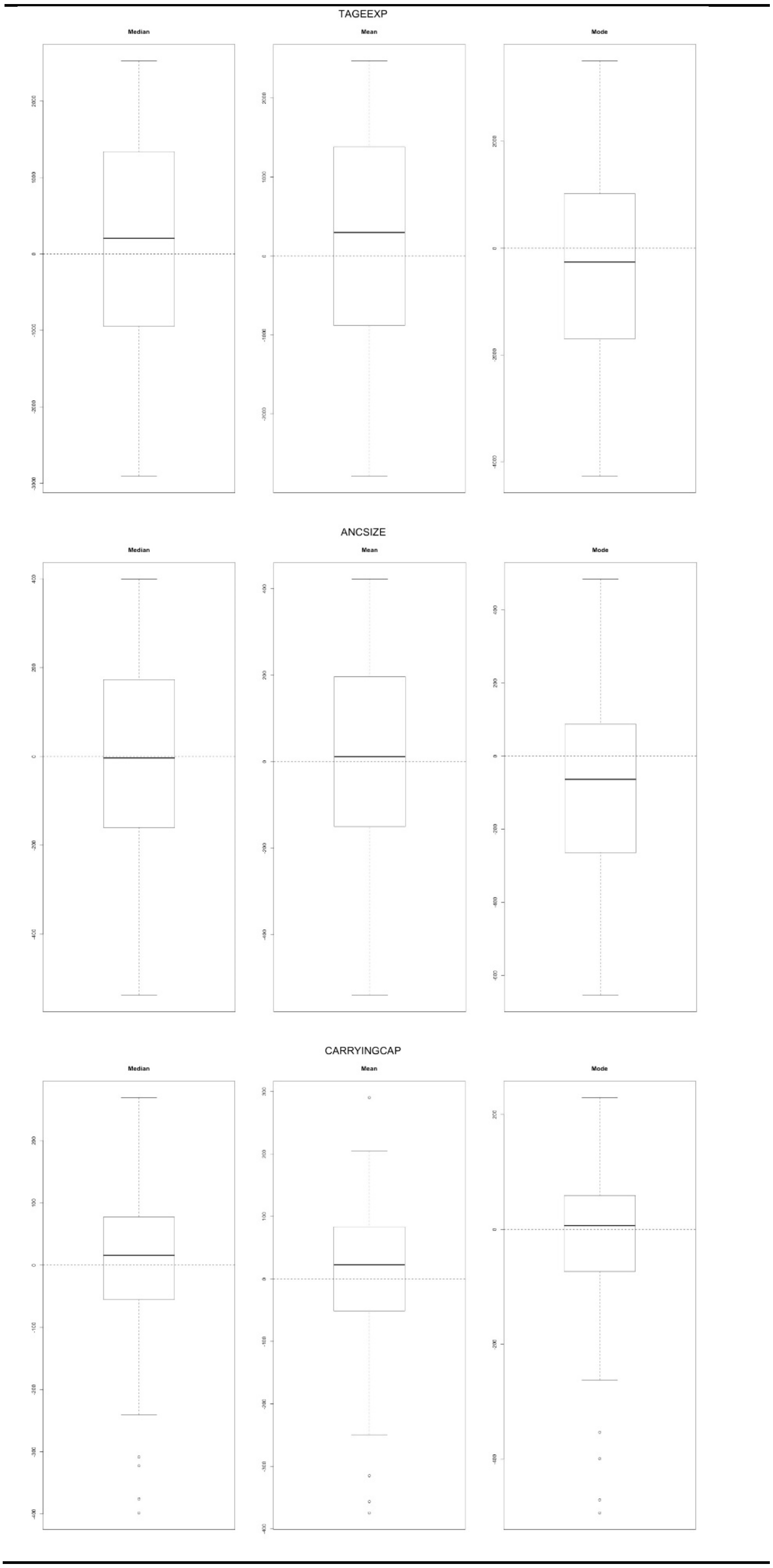
TAGEEXP
Median
Mean
Mode
ANCSIZE
Median
Mean
Mode
CARRYINGCAP
Median
Mean
Mode

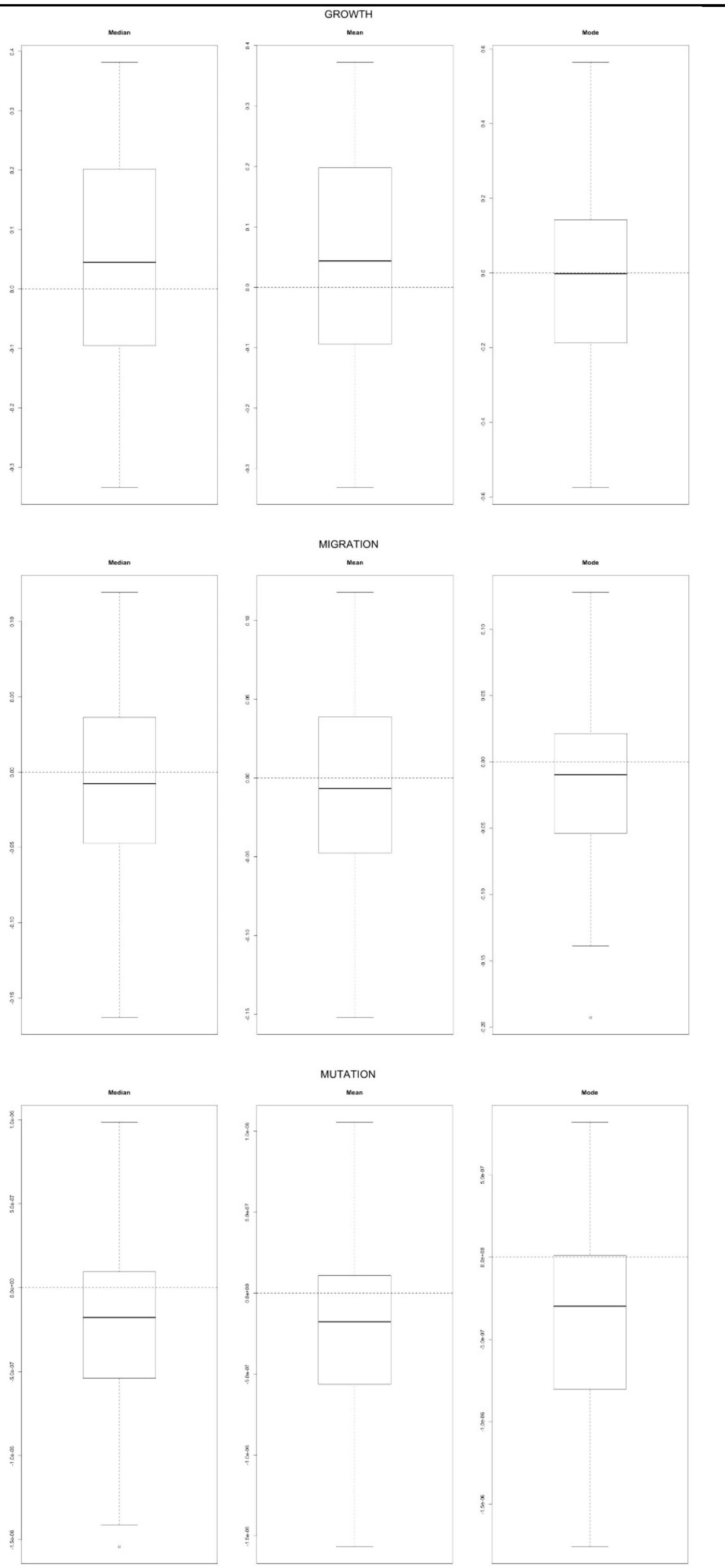

(C) Neuralnet approach

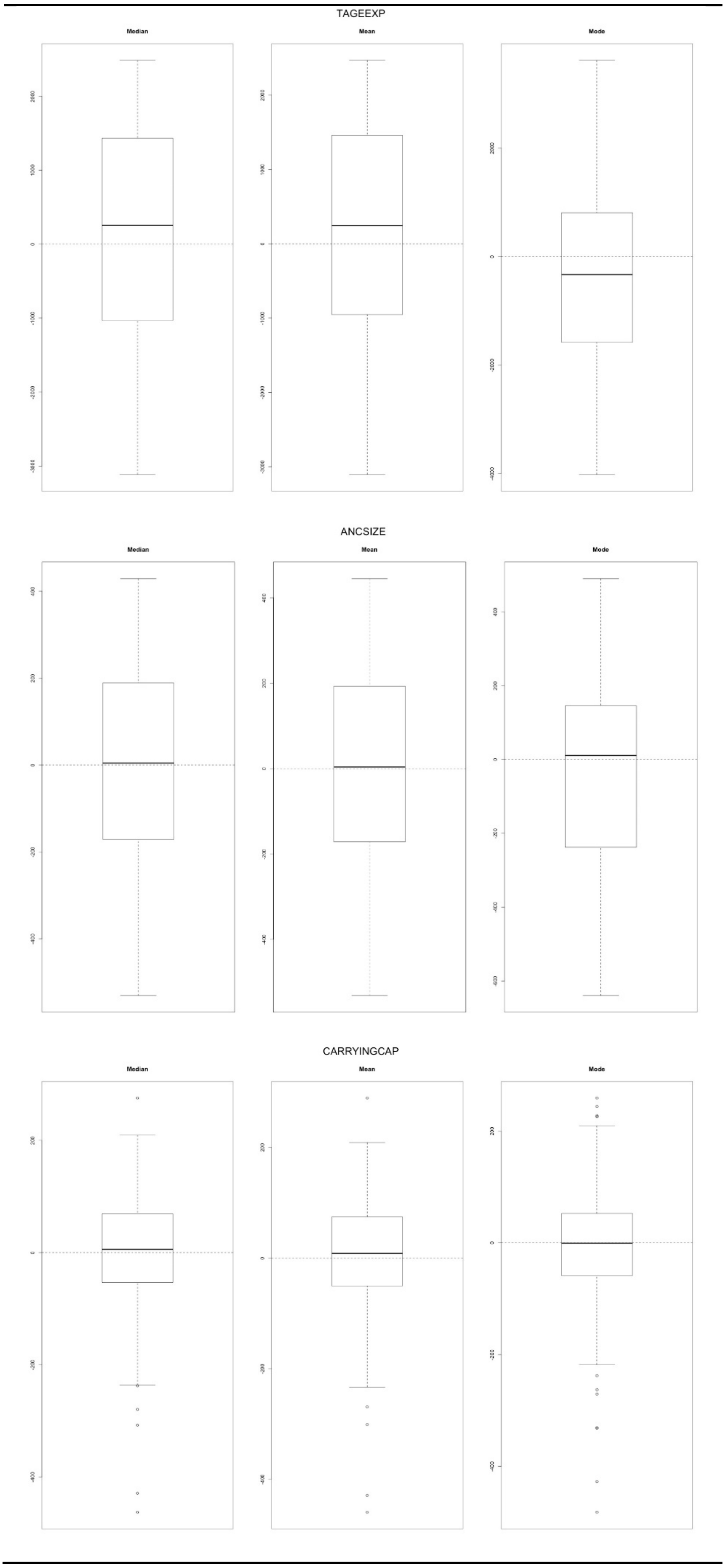
TAGEEXP
Median
Mean
Mode
ANCSIZE
Median
Mean
Mode
CARRYINGCAP
Median
Mean
Mode

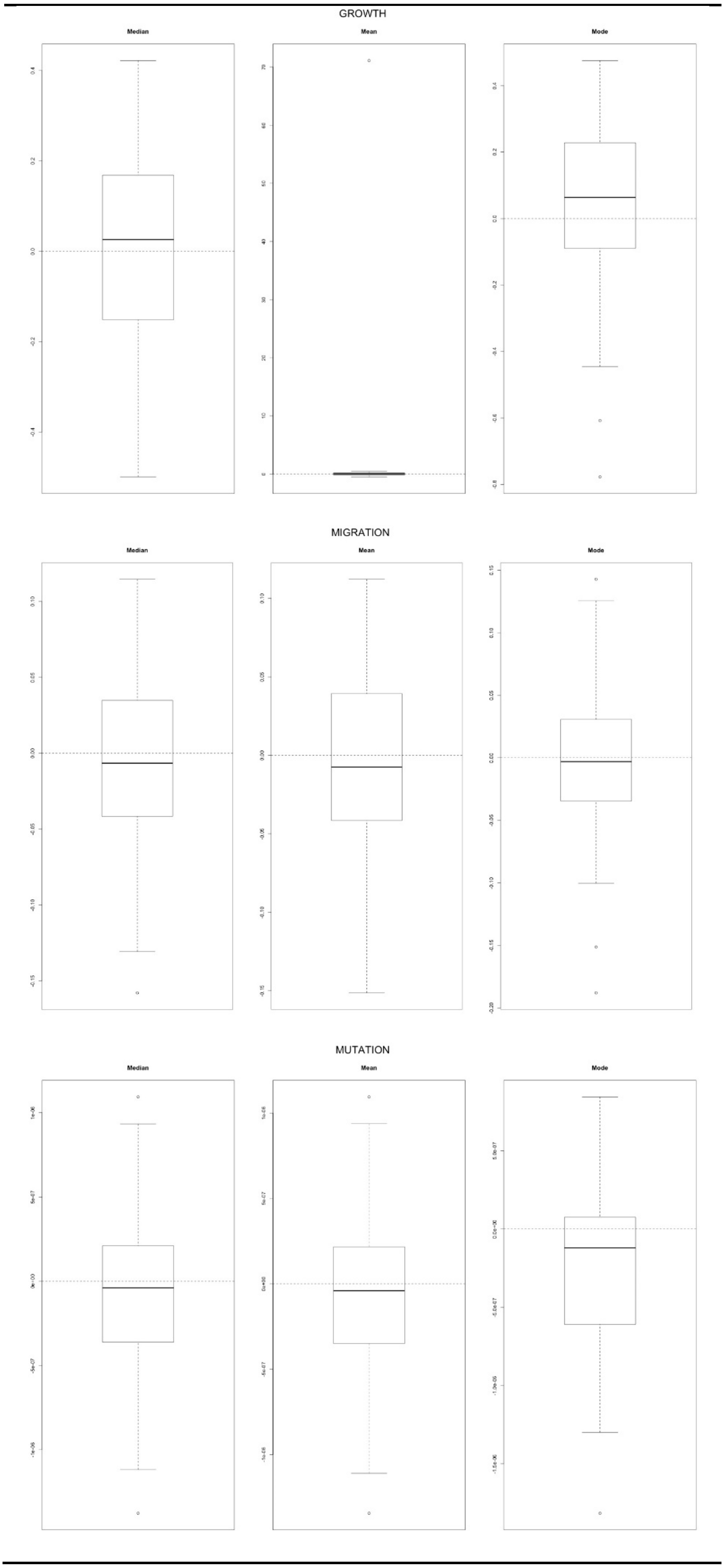
GROWTH
Median
Mean
Mode
MIGRATION
Median
Mean
Mode
MUTATION
Median
Mean
Mode

Figure 3. Power of parameter estimation. The following boxplots show, for every parameter of the best fitting model (GDD), the distance between the true (simulated) value of the POD and the estimated median, mean and mode (respectively from the left to the right). The estimations were performed with the rejection (Rrej, A), regression (Rreg, B), and neuralnet (Rnn, C) approaches implemented in the abc library of R (Csillery et al. 2012). In total, 100 PODS are used to perform this evaluation. The dashed line indicates that the true value is equal to the estimated value (error = 0). The plots show which approach and statistic of the posterior distribution is preferred for each particular parameter estimation. The time of the onset of the range expansion (TAGEEXP) was estimated with the highest accuracy by the mode of Rrej. The ancestral population size (ANCSIZE) was estimated with the highest accuracy by the mean of Rnn. The carrying capacity (CARRYINGCAP) was estimated with the highest accuracy by the mean of Rrej. The population growth rate (GROWTH) was estimated with the highest accuracy by the mode of Rreg; the migration rate (MIGRATION) was estimated with the highest accuracy by the median of Rrej. The mutation rate (MUTATION) was estimated with the highest accuracy by the mode of Rrej).

References

1. Alves, I.; Arenas, M.; Currat, M.; Sramkova Hanulova, A.; Sousa, V.C.; Ray, N.; Excoffier, L. (2016). Long-Distance Dispersal Shaped Patterns of Human Genetic Diversity in Eurasia. *Mol. Biol. Evol*. 33, 946-958.
2. Arenas, M. (2012). Simulation of Molecular Data under Diverse Evolutionary Scenarios. *PLoS Comput. Biol* 8: e1002495.
3. Arenas, M., François, O., Currat, M., Ray, N., Excoffier, L. (2013). Influence of Admixture and Paleolithic Range Contractions on Current European Diversity Gradients, *Mol. Biol. Evol.* 30: 57–61.
4. Carvajal-Rodriguez A (2010). Simulation of genes and genomes forward in time. Curr. Genomics 11: 58-61.
5. Currat, M., Ray, N., Excoffier, L. (2004) SPLATCHE: A program to simulate genetic diversity taking into account environmental heterogeneity. *Mol Ecol Notes* 4: 139–142.
6. Currat, M., Arenas, M., Quilodràn, C.S., Excoffier, K., Ray, N. (2019) SPLATCHE3: simulation of serial genetic data under spatially explicit evolutionary scenarios including long-distance dispersal. *Bioinformatics* btz311.
7. Csillery, K.; François, O.; Blum, M.G. (2012). abc: an R package for approximate Bayesian computation (ABC). *Methods Ecol. Evol* 3, 475-479.
8. Kimura M, Weiss GH (1964) The Stepping Stone Model of Population Structure and the Decrease of Genetic Correlation with Distance. *Genetics* 49: 561-576.
9. Mason, P.L., Nichols, R.A., Hewitt, G.M. (1995) Philopatry in the alpine grasshopper, *Podisma pedestris*: a novel experimental and analytical method. *Ecol. Entomol*. 20: 137-145.
10. Nakamura K, Itô, Y., Miyashita, K., Takai, A. (1964). Dispersal of adult grasshoppers *Mecostethus magister*, under the field condition. *Res. Popul. Ecol*. 6: 67-78.
11. Nei, M. (1987). *Molecular Evolutionary Genetics*. Columbia Univ. Press, New York.
12. Ray, N., Excoffier, L. (2010) A first step towards inferring levels of long-distance dispersal during past expansions. *Mol. Ecol. Res*. 10: 902-914.
13. Richards, O.W., Waloff, N. (1954) Studies on the biology and population dynamics of British grasshoppers. *Anti-locust Bull*. 17: 1-182.
14. Shapiro, L.H., Strazanac, J.S., Roderick, G.K. (2006). Molecular phylogeny of Banza (Orthoptera: Tettigoniidae), the endemic katydids of the Hawaiian Archipelago. *Mol. Phyl. Evol*. 41: 53-63.
15. Tajima, F. (1996). The amount of DNA polymorphism maintained in a finite population when the neutral mutation rate varies among sites. *Genetics* 143: 1457-1465.
16. Yang, Z. (2006). *Computational Molecular Evolution*. Oxford, England. Oxford University Press.
